# Enhancing Lymph Node Metastasis Risk Prediction in Early Gastric Cancer Through the Integration of Endoscopic Images and Real-World Data in a Multimodal AI Model

**DOI:** 10.3390/cancers17050869

**Published:** 2025-03-03

**Authors:** Donghoon Kang, Han Jo Jeon, Jie-Hyun Kim, Sang-Il Oh, Ye Seul Seong, Jae Young Jang, Jung-Wook Kim, Joon Sung Kim, Seung-Joo Nam, Chang Seok Bang, Hyuk Soon Choi

**Affiliations:** 1 Department of Internal Medicine, Seoul St. Mary’s Hospital, The Catholic University of Korea College of Medicine, Seoul 06591, Republic of Korea; etiria@catholic.ac.kr; 2 Department of Internal Medicine, Anam Hospital, Korea University College of Medicine, Seoul 02841, Republic of Korea; roadstar82@naver.com (H.J.J.); mdkorea@gmail.com (H.S.C.); 3 Department of Internal Medicine, Gangnam Severance Hospital, Yonsei University College of Medicine, Seoul 06273, Republic of Korea; xochipilli@yuhs.ac; 4 Waycen Inc., Seoul 06167, Republic of Korea; sangil.oh@waycen.com; 5 Department of Internal Medicine, Kyung Hee University Medical Center, Kyung Hee University College of Medicine, Seoul 05278, Republic of Korea; jyjang@khu.ac.kr (J.Y.J.); iloveact@hanmail.net (J.-W.K.); 6 Department of Internal Medicine, Incheon St. Mary’s Hospital, The Catholic University of Korea College of Medicine, Incheon 21431, Republic of Korea; kijoons@hanmail.net; 7 Department of Internal Medicine, Kangwon National University Hospital, Kangwon National University School of Medicine, Chuncheon 24289, Republic of Korea; pinetrees@hanmail.net; 8 Department of Internal Medicine, Chuncheon Sacred Heart Hospital, Hallym University College of Medicine, Chuncheon 24253, Republic of Korea; csbang@hallym.ac.kr

**Keywords:** stomach cancer, artificial intelligence, lymph node metastasis, clinical decision support system, multimodal artificial intelligence

## Abstract

**Objectives:** The accurate prediction of lymph node metastasis (LNM) and lymphovascular invasion (LVI) is crucial for determining treatment strategies for early gastric cancer (EGC). This study aimed to develop and validate a deep learning-based clinical decision support system (CDSS) to predict LNM including LVI in EGC using real-world data. **Methods:** A deep learning-based CDSS was developed by integrating endoscopic images, demographic data, biopsy pathology, and CT findings from the data of 2927 patients with EGC across five institutions. We compared a transformer-based model to an image-only (basic convolutional neural network (CNN)) model and a multimodal classification (CNN with random forest) model. Internal testing was conducted on 449 patients from the five institutions, and external validation was performed on 766 patients from two other institutions. Model performance was assessed using the area under the receiver operating characteristic curve (AUC), probability density function, and clinical utility curve. **Results:** In the training, internal, and external validation cohorts, LNM/LVI was observed in 379 (12.95%), 49 (10.91%), 15 (9.09%), and 41 (6.82%) patients, respectively. The transformer-based model achieved an AUC of 0.9083, sensitivity of 85.71%, and specificity of 90.75%, outperforming the CNN (AUC 0.5937) and CNN with random forest (AUC 0.7548). High sensitivity and specificity were maintained in internal and external validations. The transformer model distinguished 91.8% of patients with LNM in the internal validation dataset, and 94.0% and 89.1% in the two different external datasets. **Conclusions:** We propose a deep learning-based CDSS for predicting LNM/LVI in EGC by integrating real-world data, potentially guiding treatment strategies in clinical settings.

## Simple Summary

Artificial intelligence (AI) technology is being applied in various ways in the clinical field, with its use in medical practice rapidly expanding. This study is the first to report an integrated AI model developed for clinical decision-making in the treatment of early gastric cancer (EGC). This model combines endoscopic images with demographic data to facilitate objective clinical decision-making in real-world practice before endoscopic resection (ER) or gastrectomy. The system demonstrated consistently high performance across the training set, the internal validation set, and external validation sets from two different institutions, highlighting its strong potential for practical application. This clinical decision support system could assist physicians in making more informed decisions about ER or surgery for patients with EGC in real-world settings. Moreover, its further application has the potential to reduce medical efforts and costs by effectively identifying appropriate candidates for ER or surgery.

## 1. Introduction and Background

Gastric cancer is the fifth-most common cancer and the third leading cause of cancer-related deaths worldwide [1,2]. Endoscopic resection (ER), including endoscopic submucosal dissection (ESD), is widely accepted for effectively treating patients that have indications of tumors with a very low possibility of lymph node metastasis (LNM) and that are suitable for en bloc resection [3,4]. Thus, predicting LNM including lymphovascular invasion (LVI) ‘before’ total resection is essential to determine treatment options for early gastric cancer (EGC), such as ER or surgery.

In real-world practice, physicians have determined treatment strategies in EGC after predicting LNM risk using endoscopic images, abdominal computed tomography (CT) findings, biopsy pathology, and demographics. However, determining ER as a treatment strategy in practice can be challenging due to variability in physician experience, as the accuracy of each diagnostic method is inconsistent. There is significant interobserver variability in assessing the feasibility of ER based on endoscopic image findings, with an accuracy of only 31–38% [5,6]. Additionally, biopsy results, which represent only a portion of the lesion, can differ from the final pathological findings in nearly half of the cases [7]. CT findings for early gastric cancer are also reported to have a low sensitivity of around 48% [8]. While EUS (endoscopic ultrasonography) is a valuable diagnostic tool, it has limited reproducibility due to operator dependence, and both its sensitivity and specificity are reported to be limited [9,10,11].

As a result, approximately 16.0–20.0% of patients undergoing ER experience non-curative resection [12,13,14,15]. Of these patients, about 7.5–9.5% have LNM, which necessitates additional surgery [16,17]. For selecting patients who require radical surgery after non-curative ESD, the “eCura” system was developed [18]. However, this scoring can only be conducted after the resection of the EGC lesion because it is based on pathological findings.

With advancements in artificial intelligence (AI) technology, various models have been developed to detect and diagnose lesions. Initially, machine learning and deep learning models focused on the endoscopic morphological characteristics of lesions reported and described by endoscopists. However, recent developments enable AI models to analyze photos of the lesions themselves, offering judgment methods free from the observer’s subjectivity. Hence, this kind of clinical decision support system (CDSS) can help in determining treatment options for EGC.

AI has recently been applied in CDSSs to augment physicians’ capability to reach optimal clinical decisions [19,20]. However, until now, most CDSS models have been developed using only image or tabular data, not integrated data [19,20,21]. To select suitable patients for ER in EGC, endoscopic features are crucial for predicting the invasion depth of lesions. Thus, most studies have focused on developing AI models to predict invasion depth in EGC using endoscopic images [22,23]. The endoscopic features of EGC contain vital information about biological behaviors; therefore, many endoscopists rely heavily on them to decide whether ER should be performed. However, other clinical information, including biopsy pathology, CT findings, endoscopic ultrasound (EUS) findings, and demographic findings, is also crucial for the physician in determining treatment strategies for EGC. According to a previous study, abnormal CT findings such as gastric fold thickening or reactive LN enlargement are significantly related to non-curative resection after ER [24].

Therefore, developing an AI model that integrates endoscopic images with other clinical information, similar to in a real-world situation, can prove to be a more useful CDSS in EGC. This study aims to develop and validate a deep learning-based CDSS for predicting LNM including LVI in EGC by integrating real-world data before resection.

## 2. Methods

### 2.1. Study Design and Data Preparation

**Figure 1:**
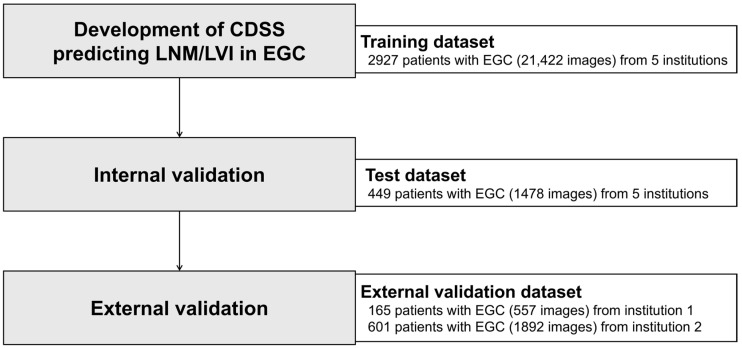
Flowchart of the study design. CDSS—clinical decision support system; EGC—early gastric cancer; LNM—lymph node metastasis; LVI—lymphovascular invasion.

This study was conducted across multiple centers (Figure 1), involving seven different institutions. Data from five institutions (Seoul St. Mary’s Hospital, Incheon St. Mary’s Hospital, Gangnam Severance Hospital, Korea University Anam Hospital, and Gangwon University Hospital) were used for the training and testing datasets, and data from two other institutions were used for external validation (Chuncheon Sacred Heart Hospital for external validation 1; Kyung Hee University Medical Center for external validation 2).

Patients diagnosed with EGC were enrolled in this study. All patients underwent curative treatment with either surgery or ER between January 2010 and December 2015. Patients who underwent ER were enrolled after ascertaining the absence of five-year recurrence, because LNM was not pathologically confirmed. No recurrence over five years post-ER was clinically regarded as no LNM. The endoscopic images and clinical information of each patient were obtained. Clinical information included tabular data that could be obtained before resection, such as age, sex, tumor location, size, biopsy pathology, and CT descriptions provided by expert radiologists (unremarkable finding; gastric fold thickening; LN enlargement including reactive, metastatic suspicious LN). For the ground truth, pathological results from resected specimens including the pathological diagnosis, tumor location and size, invasion depth, LNM, and LVI were also collected.

Endoscopic static images were obtained using standard endoscopes (GIF-Q260J, GIF-H260, and GIF-H290; Olympus Medical Systems Co. Ltd., Tokyo, Japan). The endoscopic images included only white light images. In addition, poor-quality images, such as those that were out of focus or those with motion blurring, halation, or poor air insufflation, were excluded. In addition, patients lost to follow-up after ER or in whom LN recurrence was observed after ER were excluded.

Tabular patient data, including demographic data, biopsy pathology, and CT findings, were also collected. Multiple endoscopic images and tabular data were integrated for each patient. Cases with missing data were excluded. This study was approved by the institutional review boards of each institution (institutional review board of Seoul St. Mary’s Hospital and Incheon St. Mary’s Hospital (XC22RCDI0068), institutional review board of Korea University Anam Hospital (2022AN0013), institutional review board of Gangnam Severance Hospital (3-2021-0330), institutional review board of Gangwon National University Hospital (KNUH-2021-10-014-003), institutional review board of Kyung Hee University Hospital (KHUH 2021-09-016), and institutional review board of Chuncheon Sacred Heart Hospital (2022-03-022)).

### 2.2. Model Construction

We utilized the data of 2927 patients with EGC from five institutions as a training set to develop the CDSS to predict LNM/LVI before total resection.

To increase the diversity of data representations and decrease data scale bias caused by the imbalance between classes, we employed image data augmentation methods during the training time. For data of a class with a relatively small size of image sets, randomly generated images by augmentation methods were additionally trained. Flipping, rotation, blur, and Gaussian noises were applied by random parameters to the negative class 1 time because the number of images in the positive class (13,167 images from 545 patients) was larger by about 1.6 times than that in the negative class (8255 images from 2382 patients).

### 2.3. Image-Only-Based Model: Basic Convolutional Neural Network (CNN)

**Figure 2:**
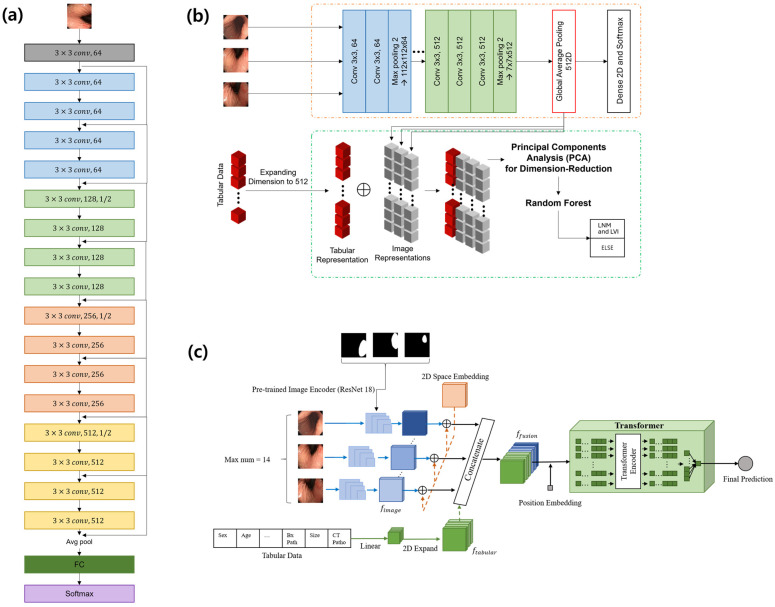
Mechanisms of models. (**a**) Basic CNN model; (**b**) CNN with random forest model; (**c**) transformer-based model. CNN: convolutional neural network.

We trained a model using endoscopic images to predict LNM/LVI. These models were composed of fully connected layers with multiple neural units after consecutive convolutional blocks. For the CNN-based predictor, ResNet18, which has shown significant success in numerous studies, was used (Figure 2a). ResNet18 has been used for various computer vision tasks including in the medical domain with stable performance, because the number of layers that the architecture consists of is sufficient for learning a dataset of a moderate size. To explore the performance of various CNN architectures on the image-only task, we compared them, as shown in the Supplementary Materials. The models were pre-trained on a dataset for an endoscopic lesion detection task, and transfer learning was utilized. All pre-trained convolutional layers were fixed, whereas the fully connected layers and the final classification layer were fine-tuned on the new task. As a result, the accuracies of VGG16 and VGG19 are 58.57% and 52.12%, respectively, whereas the ResNet18 shows 61.92% accuracy (Supplementary Table S1).

### 2.4. Multimodal Classification Model: CNN with Random Forest

Utilizing only a single data modality to predict LNM/LVI in patients with EGC is ineffective because of the wide range of available data. Therefore, utilizing information from multimodal data is important for improving the performance of target tasks. Through the use of a CNN, the task could be favorably trained in the end-to-end process. However, there are some limitations to simultaneously handling and fusing multimodal data. Specifically, controlling the scale and dimension imbalances between input multimodal data is too parametric to stabilize them. Therefore, to efficiently secure the optimized performance and extract intact fusion information, we built a two-stage predictor. In this study, we developed a traditional fusion model to jointly use multimodal data, including multiple endoscopic images and tabular data. The model consisted of a CNN for image features and a fusion module.

The CNN architecture and training strategy for extracting image features were based on the image classifier *v*2 (IC *v*2), which was designed to predict the invasion depth of EGC from an endoscopic image [22]. To jointly use the image features and tabular data, a random forest model and principal component analysis were constructed for fusing multiplicative factors (Figure 2b). The details of the model and the preparation process to fuse the multimodal data are described in the Supplementary Materials.

### 2.5. Transformer-Based Model

To predict LNM/LVI by considering both tabular and image data, we constructed another model architecture based on the transformer [25]. Although there have been attempts to combine multimodal data, these methods have been based on CNN-based modules which have a receptive field with a limited range. The scale of the receptive field for input data is closely associated with the ability to catch hidden information in the multimodal features. By overcoming these challenges, the transformer has shown comparable performance in a wide range of machine learning fields such as natural language processing and computer vision. In addition, the nature of the transformer in self-attending to relations between multiple modalities allows for significant achievements in multimodal data. The model follows the encoder–predictor routine, including two separate unimodal encoders for the image and tabular data, and a transformer-based predictor for fusing feature representations from multimodal data. An overview of the model is presented in Figure 2c. The maximum number of input images which the encoder for the image inputs takes is 14. This setting was heuristically chosen from the effectiveness experiments of validated ranges: if the number was increased up to 15, a performance decrease was observed. While the unimodal encoders extract feature representations from each modality, the transformer integrates them to predict LNM/LVI. The architecture and input–output constructions are described in the Supplementary Materials.

### 2.6. Internal and External Validations

The model was internally validated using data from 449 mutually exclusive patients from the first five institutions (Seoul St. Mary’s Hospital, Incheon St. Mary’s Hospital, Korea University Anam hospital, Gangnam Severance Hospital, and Kangwon University Hospital). These patients’ data did not overlap with the data used for model construction. External validation was performed using data from two independent institutions (Chuncheon Sacred Heart Hospital and Kyung Hee Medical Center), comprising 165 and 601 patients, respectively. To verify the model’s consistency across different cohorts, external validation was conducted separately using data from two institutions.

### 2.7. Outcome

The endpoint selected to develop the AI models was predicting LNM/LVI requiring surgery. This is considered to be the most useful outcome for identifying patients suitable for ER alone. The sensitivity, specificity, positive predictive value (PPV), negative predictive value (NPV), and area under the receiver operating characteristic curve (AUC) were used to compare the performance of the developed model, including the basic CNN, CNN with random forest, and transformer-based models. The best model based on these parameters was selected and validated.

### 2.8. Statistical Analyses

ANOVA tests for continuous variables and chi-square tests for categorical variables were used to compare baseline characteristics between the training and validation sets. The performance of the developed model was evaluated by calculating the AUC. Our primary analysis involved comparing the AUCs for each constructed AI model, followed by a secondary analysis that included the calculation of parameters, such as the AUC, sensitivity, specificity, PPV, and NPV, of the best-performing model using two different external validation datasets. In addition, the predictive performance was assessed by the probability density function (PDF) and clinical utility curve (CUC) to determine the clinical utility thresholds [26].

## 3. Results

### 3.1. Patient Characteristics

The baseline characteristics of the training, internal validation, and external validation sets are summarized in Table 1 and Table 2. Tabular characteristics including location, pathologic diagnosis, tumor size, invasion depth, CT findings, LVI, and LNM were similar between the training and internal validation sets. The proportion of LNM/LVI was 12.95% in the training set, 10.34% in the internal validation set, 8.67% in external validation set 1, and 6.60% in external validation set 2.

### 3.2. Selection of Best-Performing Model

**Figure 3:**
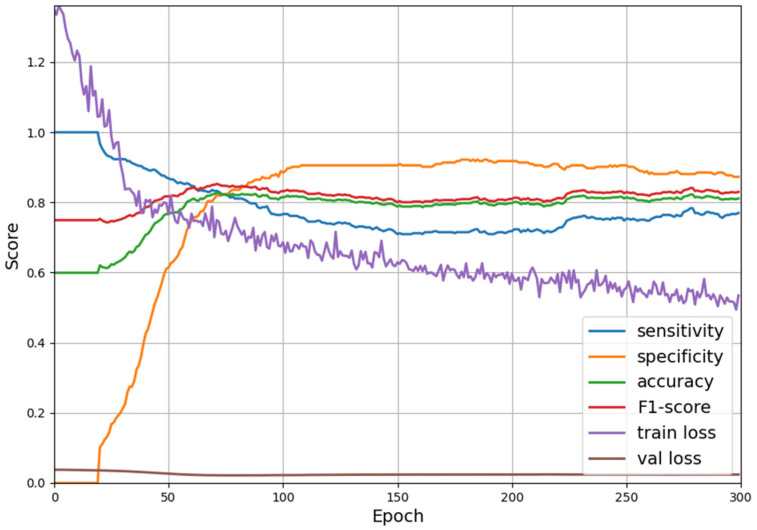
The training convergence process of the transformer-based model in recall scores.

Table 3 presents the results of the three models. The basic CNN model achieved an AUC of 0.5937 with 53.06% sensitivity and 63.00% specificity, and the CNN with random forest model achieved an AUC of 0.7548 with 67.35% sensitivity and 74.50% specificity. The transformer-based model achieved the highest AUC (0.9083) with comparable sensitivity (85.71%) and specificity (90.57%). The PPV (89.74%) and NPV (86.59%) were also the highest. The convergence process of the selected model is described in Figure 3. The sensitivity, specificity, accuracy, F1-score, and val loss were computed on the training–validation set, in which 10% of the data were randomly sliced and taken from the training dataset.

### 3.3. Outcomes of Internal and External Validation

The internal validation group comprised 449 randomly selected patients. Their demographic characteristics are summarized in Table 2. The basic CNN model had the lowest AUC value (0.5937, 95% CI 0.5483–0.6392), followed by the CNN with random forest model (0.7548, 95% CI 0.7151–0.7946) and the transformer-based model (0.9083, AUC 0.8816–0.9350). The transformer-based model exhibited the highest sensitivity (85.71%), specificity (90.75%), PPV (89.74%), and NPV (86.59%). The results are summarized in Table 3.

External validation was conducted using the transformer-based model on two datasets from different institutions. These two datasets differ, particularly in terms of patient age, lesion location, and lesion size (Table 2). In the first external validation set, a high AUC of 0.9404 was observed with comparable sensitivity (93.33%), specificity (97.33%), PPV (97.22%), and NPV (93.59%). In the second external validation set, the AUC remained high (0.8906), with comparable sensitivity (87.80%), specificity (89.11%), PPV (89.20%), and NPV (87.70). The transformer-based model showed consistent performance in two different validation cohorts, implying high versatility. The results are summarized in Table 4.

**Figure 4:**
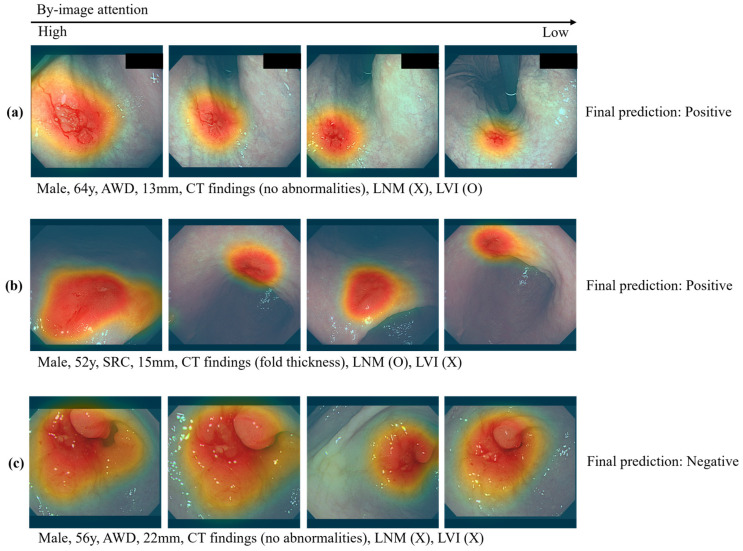
The attention ranks inferred by the transformer-based model. Each row is images from a patient, whereas each column is the attention rank between images. The heatmap was extracted by using the Grad-CAM method. In the heatmap, the red color means high attention for the 2D image space. (**a**) A case predicted as positive by the transformer-based model: A 64-year-old male with AWD and a 13 mm lesion, showing normal findings on CT. The actual result was LVI-positive, which matched the prediction. (**b**) A case predicted as positive: A 52-year-old male with SRC and a 15 mm lesion, showing fold thickness on CT. The actual result was LNM-positive, which matched the prediction. (**c**) A case predicted as negative: A 56-year-old male with AWD and a 22 mm lesion, showing normal findings on CT. The actual results were LNM-negative and LVI-negative, which matched the prediction.

Example result images are shown in Figure 4. The first two rows are positive cases, while the last row is a negative case. The images in the left column have a higher attention rank than those on the right side. For the tabular data, the CT findings had the highest attention rank, and the biopsy results followed.

### 3.4. Choice of the Best Threshold Probability for Clinical Utility

**Figure 5:**
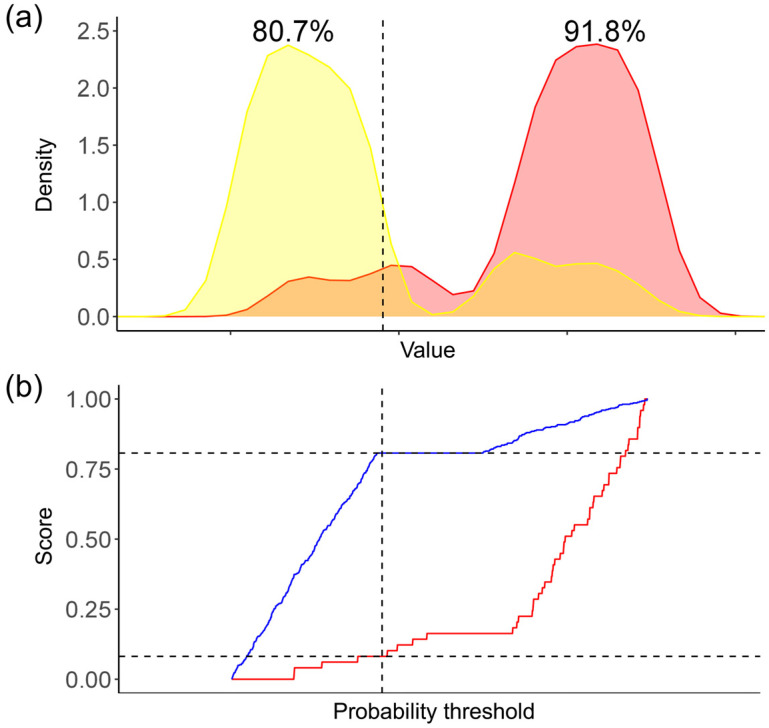
Probability density function and clinical utility curve. (**a**) Probability density function. (**b**) Clinical utility curves to decide clinical utility thresholds suggested 36.2% as a threshold probability for guiding the diagnosis of lymph node metastasis (LNM) or lymphovascular invasion (LVI), which could distinguish approximately 91.8% of patients with LNM/LVI, 80.0% (external validation set 1), and 85.4% (external validation set 2).

The PDF of the LNM test group is shown in Figure 5a, and the CUC, which explains the percentage of detected LNM/LVI-positive patients at any probability threshold, is shown in Figure 4b. Based on these results, from a clinical perspective, we chose 36.24% as the threshold probability for making clinical decisions using Youden’s J statistic. Using this threshold, we were able to distinguish 91.8% of patients with LNM/LVI in the internal validation set, which is considered an acceptable determination rate. For the external validation sets, 94.04% and 89.06% of patients with LNM/LVI could be distinguished, respectively.

## 4. Discussion

We developed a CDSS to predict LNM/LVI ‘before’ resection, mirroring real-world scenarios. Although endoscopic images can be the most important in deciding upon ER or surgery, most physicians consider demographic data and CT results to determine whether to proceed with ER or surgery, reflecting the real clinical environment.

Our CDSS model is the first to integrate endoscopic images with tabular information in EGC, similar to real-world situations. We also incorporated up-to-date transformer models that showed comparable results, including a high AUC and sensitivity, in both the internal validation set and two different external validation sets. This innovative approach could assist physicians in making optimal decisions, regardless of their level of experience.

The transformer was originally used to train long-range dependencies in sequence-to-sequence language tasks. Recently, transformer-based vision models have demonstrated state-of-the-art performance in vision tasks because of their ability to learn dense correlations between tokens. Transformers have been shown to competitively outperform existing tasks and models by dividing dense, continuous signals into subpatches and rasterizing them into 1D tokens. This early fusion model allowed us to freely learn the attention flow between different spatiotemporal regions in the image as well as across modalities.

Applications of AI systems have been widely explored in many medical fields, and these systems have achieved significant performance growth in their tasks. Contrary to other applications and studies utilizing medical image data, however, securing and gathering relevant datasets is more difficult in endoscopy than in other data modalities such as CT and MRI due to the nature of its data acquisition. Specifically, if the patient pool for the target task is narrow, constructing a robust dataset is impractical in terms of class imbalance. Therefore, extracting core information from limited representations is too difficult in building a model that can successfully conduct target tasks. This challenge emphasizes the necessity of combining multimodal data to gain insight from multifarious representations of information. However, optimizing all data modalities with a single objective optimizer can lead to overfitting to a specific modality due to dimension imbalance. In this paper, we have observed that the used model architecture can effectively classify and predict LNM/LVI by alleviating the lack of a data pool, class imbalance, and dimension imbalance.

In a clinical environment, physicians may encounter challenges when deciding whether a patient should undergo ER. The endoscopic appearance and results of EUS can assist in assessing the depth of a lesion [27], which is crucial for considering curative resection. Despite reports suggesting that EUS does not significantly change pretreatment T staging in patients with EGC compared with conventional endoscopy [10], it can still be a valuable tool. Nevertheless, for doctors, determining the T stage using only endoscopic visualization is challenging. Various models assisted by AI technology have been developed to ascertain lesion characteristics using endoscopic images. A well-designed AI model can outperform expert endoscopists in accurately predicting the depth of invasion of the lesion [28] and delineating the line of tumors using magnifying narrow-band endoscopy images [29]. However, in our study, the basic CNN model showed questionable results for predicting LNM risk, with a low AUC (0.5937) and sensitivity (53.06%).

CT was used to evaluate the N and M stages before treatment. A deep learning-based radiomic nomogram has demonstrated predictive capabilities for LNM in locally advanced gastric cancer with wall invasion beyond the submucosa [30]. Another study developed a deep learning model using data from patients who had undergone gastrectomy to predict LNM [31], achieving an accuracy of 90%. One study also created a predictive model for the occurrence of LNM in patients with EGC who underwent radical gastrectomy, reporting an AUC of 0.834 [32]. However, these studies are not clinically applicable because they included a range of T stages, did not examine endoscopic images which are vital for deciding the treatment approach, and were not externally validated.

A study reviewing the records of patients with EGC who underwent curative surgery suggested that the expanded criteria for ER could be acceptable in cases without LNM [33]. However, a minimal risk of LNM still exists. In our study, approximately 20% of the subjects in the training dataset who underwent ER and met the expanded criteria (344 of 1608 patients, 78.6%) were found to have LVI or LNM after ER and needed to undergo surgery. Our model outperformed classical physician experience-based decisions in both the training and validation sets. Using the transformer-based model, we successfully distinguished 90.83% of the EGC patients with LNM/LVI. Furthermore, this result was consistent across the two different validation sets, with the AUC exceeding 0.80 in both sets (0.9258 and 0.8506).

Sensitivity is an important parameter in clinical decision-making to reduce the probability of insufficient treatment for gastric cancer. The transformer-based model achieved a sensitivity of 80% or higher for all datasets, suggesting the potential clinical utility of this CDSS for EGC treatment. Moreover, the PPVs were high across all datasets, indicating the high utility of the model.

Our study has several strengths in various aspects. First, it examined both endoscopic images and demographic data, which are used for clinical decision-making in real world clinical practice, without subjectivity. With the aid of an AI model, physicians can make clinical decisions with a certain degree of accuracy regardless of their experience. To the best of our knowledge, this is the first study to report an integrated AI model developed for clinical decision-making in EGC treatment based on predicting LNM/LVI. Second, compared to previous studies, this study included a large number of patients from multiple institutions. This large number of subjects and institutions may help minimize bias. Additionally, the characteristics of the three validation sets were different; however, the transformer-based model still demonstrated good performance. This suggests that the model has addressed a crucial AI model development challenge: overfitting. The model exhibited consistent performance across different datasets, implying the possibility of its general use in various clinical fields. Third, our dataset comprised surgical cases and patients who underwent ER. We inferred that if a patient did not experience recurrence within five years after ER, LNM could be considered negative. This assumption holds greater relevance in real-world scenarios.

The limitations of this study are as follows: First, the model was developed based on data from a single ethnicity—Koreans. However, owing to the high incidence of gastric cancer in Korea and other countries, this model still has high utility for deciding treatment options in real-world situations. Second, the findings from the CT scans were included in the model as categorized variables, although the results of CT scans are somewhat measurable compared to endoscopic findings. This suggests the need for further research that includes the images themselves for more objective model construction. Third, in our study, patients who experienced recurrence after ESD were excluded. For this group, it is difficult to distinguish whether recurrent lesions are due to initial LNM or if they occur metachronously. Therefore, we excluded these subjects in order to minimize potential bias. Fourth, our study does not provide a head-to-head comparison of LNM/LVI prediction between physicians and a transformer-based AI model. However, since this study evaluates the performance of an AI model and demonstrates its potential application in clinical practice, it is deemed possible to conduct a study comparing it with expert opinions.

Despite these limitations, our study is the first to develop and validate the utility of the CDSS for predicting LNM/LVI in EGC by integrating real-world data. This CDSS can provide practical support to physicians in real clinical settings when deciding between ER or gastrectomy for patients with EGC. Notably, while such decisions were previously influenced by the physician’s experience and skill, the assistance of AI enables the determination of treatment strategies to be more objective. The further application of this system may reduce medical effort and costs by selecting appropriate candidates for ER or surgery. Through broader applications, this model can evolve and be used more widely in clinical practice.

## 5. Conclusions

The newly developed and validated transformer-based CDSS integrating endoscopic images and demographic data to predict LNM/LVI in EGC achieved high accuracy and sensitivity across diverse datasets. By providing objective and consistent support for treatment decisions, this AI model has the potential to enhance clinical decision-making, reduce reliance on physician experience, and optimize patient outcomes in real-world practice. Further studies incorporating various images themselves for a more objective model and including diverse ethnic groups are expected to contribute significantly to the treatment of patients with EGC.

## Figures and Tables

**Table 1:** Demographic findings of the training set.

	Training Set (N = 2927)
Age (yr, mean ± SD)	63.1 ± 11.3
Sex (male, n, %)	1985 (67.8)
Location (n, %)	
Upper 1/3	289 (9.9)
Mid 1/3	889 (30.4)
Lower 1/3	1749 (59.7)
Biopsy pathology (n, %)	
AWD	1141 (39.0)
AMD	983 (33.6)
APD	426 (14.5)
SRC	377 (12.9)
Size (mm, mean ± SD)	24.6 ± 16.7
Invasion depth (n, %)	
Mucosa	1879 (64.4)
Submucosa	1039 (35.6)
CT findings (n, %)	
No abnormalities	2133 (72.9)
Stomach thickening	430 (14.7)
Reactive LN	184 (6.3)
Metastatic LN	22 (0.8)
Stomach + LNM	158 (5.4)
LNM/LVI (n, %)	379 (12.9)
LNM (n, %)	233 (8.0)
LVI (n, %)	312 (10.7)
Both (n, %)	166 (5.7)

SD—standard deviation; AWD—adenocarcinoma well differentiated; AMD—adenocarcinoma moderately differentiated; APD—adenocarcinoma poorly differentiated; SRC—signet ring cell carcinoma; LN—lymph node; LNM—lymph node metastasis; LVI—lymphovascular invasion.

**Table 2:** Demographic findings of the validation sets.

	Internal Validation (N = 449)	External Validation 1 (N = 165)	External Validation 2 (N = 601)	*p*-Value
Age (yr, mean ± SD)	65.2 ± 11.47	76.17 ± 11.22	64.51 ± 10.31	<0.001
Sex (male, n, %)	312 (69.5)	112 (67.9)	410 (68.2)	0.885
Location (n, %)				<0.001
Upper 1/3	27 (6.0)	12 (7.3)	60 (10.0)	
Mid 1/3	131 (29.2)	37 (22.4)	237 (39.4)	
Lower 1/3	291 (64.8)	116 (70.3)	304 (50.6)	
Biopsy pathology (n, %)				0.071
AWD	196 (43.7)	61 (37.0)	244 (40.6)	
AMD	160 (35.6)	65 (39.4)	195 (32.4)	
APD	45 (10.0)	26 (15.8)	81 (13.5)	
SRC	48 (10.7)	13 (7.9)	81 (13.5)	
Size (mm, mean ± SD)	22.34 ± 14.12	3.02 ± 3.55	18.6 ± 11.57	<0.001
Invasion depth (n, %)				
Mucosa	317 (70.6)	118 (72.4)	464 (77.2)	
Submucosa	132 (29.4)	45 (27.6)	137 (22.8)	
CT findings (n, %)				<0.001
No abnormalities	306 (68.5)	153 (92.7)	526 (87.5)	
Stomach thickening	88 (19.7)	8 (4.9)	42 (7.0)	
Reactive LN	31 (6.9)	0 (0)	30 (5.0)	
Metastatic LN	3 (0.7)	2 (1.2)	1 (0.2)	
Stomach + LNM	19 (4.3)	0 (0)	2 (0.3)	
LNM/LVI (n, %)	49 (10.3)	15 (8.7)	41 (6.6)	0.083
LVI (n, %)	36 (8.0)	11 (6.7)	28 (4.7)	0.079
LNM (n, %)	37 (8.2)	4 (2.4)	16 (2.7)	<0.001
Both (n, %)	24 (5.3)	0 (0)	3 (0.5)	<0.001

SD—standard deviation; AWD—adenocarcinoma well differentiated; AMD—adenocarcinoma moderately differentiated; APD—adenocarcinoma, poorly differentiated; SRC—signet ring cell carcinoma; LN—lymph node; LNM—lymph node metastasis; LVI—lymphovascular invasion.

**Table 3:** Performance comparison of the basic CNN model, the CNN with random forest model, and the transformer-based model on the internal validation set.

	Accuracy (%)	Sensitivity (%)	Specificity (%)	PPV (%)	NPV (%)	F1-Score (%)	AUC
Basic CNN	61.92 (57.42–66.41)	53.06 (39.09–67.03)	63.00 (58.27–67.73)	58.43 (43.95–72.91)	57.80 (44.69–70.91)	55.61 (41.37–69.85)	0.5937 (0.5483–0.6392)
CNN with random forest	73.72 (69.65–77.79)	67.35 (54.22–80.48)	74.50 (70.23–78.77)	72.13 (59.14–85.12)	69.95 (57.64–82.27)	69.66 (56.57–82.73)	0.7548 (0.7151–0.7946)
Transformer-based	91.31 (88.71–93.92)	89.80 (81.32–98.27)	91.50 (88.77–94.23)	91.19 (83.19–99.19)	90.15 (81.95–98.35)	90.49 (82.25–98.73)	0.9182 (0.8929–0.9436)

CNN—convolutional neural network; PPV—positive predictive value; NPV—negative predictive value; AUC—area under the receiver operating characteristic curve.

**Table 4:** Results of transformer-based model on validation sets.

	Accuracy (%)	Sensitivity (%)	Specificity (%)	PPV (%)	NPV (%)	F1-Score (%)	Macro F1-Score (%)	AUC
Internal validation	91.31 (88.71–93.92)	89.80 (81.32–98.27)	91.50 (88.77–94.23)	91.19 (83.19–99.19)	90.15 (81.95–98.35)	90.49 (82.25–98.73)	82.12	0.9182 (0.8929–0.9436)
External validation 1	96.97 (94.35–99.59)	93.33 (80.71–100.00)	97.33 (94.76–99.91)	97.22 (88.73–100.00)	93.59 (81.44–100.00)	95.25 (84.53–100.00)	91.58	0.9404 (0.9043–0.9766)
External validation 2	89.02 (86.52–91.52)	87.80 (77.79–97.82)	89.11 (86.53–91.69)	89.20 (79.63–98.78)	87.70 (77.60–97.80)	88.50 (78.70–98.30)	72.99	0.8906 (0.8657–0.9156)
Mean (standard deviation)	92.43 (3.341)	90.31 (2.286)	92.65 (3.452)	92.54 (3.410)	90.48 (2.416)	91.41 (2.832)	82.23 (7.590)	0.9164 (0.0203)

PPV—positive predictive value; NPV—negative predictive value; AUC—area under the receiver operating characteristic curve.

## Data Availability

The data presented in this study are available on request from the corresponding author due to personal information protection.

## Supplementary Materials

The following supporting information can be downloaded at https://www.mdpi.com/article/10.3390/cancers17050869/s1, Table S1: Performances of the models.
