# Supplementary material for: Enhancing Lymph Node Metastasis Risk Prediction in Early Gastric Cancer Through the Integration of Endoscopic Images and Real-World Data in a Multimodal AI Model
Source: Cancers (Basel). 2025 Mar 3;17(5):869. doi: 10.3390/cancers17050869 (PMC11898873; doi:10.3390/cancers17050869)
Supplement: Supplementary file 1 [file cancers-17-00869-s001.zip › cancers-3311557-supplementary.pdf]

Supplementary Materials

Multimodal classification model: CNN with random forest

Typically, EGC patients have more than one endoscopic image capturing the EGC lesions. To reflect the various viewpoints of the lesion in the model, we used all endoscopic images of a patient including the target EGC lesion. After feeding each endoscopic image to IC $v2$[1], the output of the global average pooling layer was extracted as an image feature representation (512-dimension).

The tabular data included numerical and categorical fields. We quantized the numerical values (age and lesion size) into categorical form. The age field was categorized based on being 60 years old (e.g., 0 for under 60 years, and 1 for 60 years old or more), while the lesion size was based on the 20 mm. To jointly use image and tabular representations by concatenating them, we stretched the tabular representation to 512 dimensions using a fully connected layer. Finally, the dimensions of the concatenated representations were reduced for use as a dense input to the random forest model using principal component analysis.

Transformer-based model

The image encoder uses the consecutive convolutional blocks to extract a set of 2D representations $\mathcal{F}_{i\in I_N} \in \mathbb{R}^{H_\mathcal{F}\times W_\mathcal{F}\times C_\mathcal{F}}$ from all input endoscopic images $x_{i\in I_N} \in \mathbb{R}^{H\times W\times C}$. Each image is fed into the network sequentially. The feature maps of each image generated from the CNN encoder can be described as $\mathcal{F}_i^{image} = f^{conv}(x_i; \theta^{conv})$, where θ^{conv} denotes the trained parameters of the image encoder. In this study, we used a set of convolutional blocks from ResNet18 as the image encoders.

Concatenating image and tabular representations is essential for using them jointly as input for the transformer-based predictor. However, because of mismatched dimensions, direct concatenation of the tabular data x_T and its feature representations with image representations is not feasible. Therefore, to align these dimensions, we expanded the dimensions of the tabular representations ($\mathcal{F}_T$) to a 2D matrix-

like structure by tiling each component ($H_{\mathcal{F}} \times W_{\mathcal{F}} \times \mathcal{D}_T$) after linearly transforming the original tabular shapes ($1 \times 1 \times \mathcal{D}_T$). As a result, the transformed tabular representation can be denoted as $\mathcal{F}_T \in \mathbb{R}^{H_{\mathcal{F}} \times W_{\mathcal{F}} \times \mathcal{D}_T}$.

Since the transformer considers the input in a sequential format to model serially correlated information, we fed the channel-wise stacked feature representations as a sequentially fused input $\mathcal{F}_{fuse} \in \mathbb{R}^{H_{\mathcal{F}} \times W_{\mathcal{F}} \times ((C_{\mathcal{F}} \times I_N) + \mathcal{D}_T)}$. At this point, a position embedding procedure was attached to the input stage to allow the transformer to learn the sequential order of the fused representation. There was no significant correlation between the order of the input endoscopic images. Therefore, we randomly shuffled the input bunch of endoscopic images while training the model. The transformer receives the fused representations and outputs as a $(C_{\mathcal{F}} \times I_N) + \mathcal{D}_T$-dimensional vector. Subsequently, the final probability of LNM and/or LVI was predicted by applying a fully connected layer to the Softmax activator.

Supplementary references

1. Kim J-H, Oh S-I, Han S-Y, et al. An optimal artificial intelligence system for real-time endoscopic prediction of invasion depth in early gastric cancer. *Cancers* 2022;14:6000.
